# Methodological approach for measuring the effects of organisational-level interventions on employee withdrawal behaviour

**DOI:** 10.1007/s00420-021-01686-y

**Published:** 2021-03-26

**Authors:** M. Akerstrom, J. Severin, H. Imberg, I. H. Jonsdottir, L. Björk, L. Corin

**Affiliations:** 1 Region Västra Götaland, Institute of Stress Medicine, Gothenburg, Sweden; 2 Occupational and Environmental Medicine, School of Public Health and Community Medicine, Institute of Medicine, The Sahlgrenska Academy at University of Gothenburg, Gothenburg, Sweden; 3 Department of Mathematical Sciences, Chalmers University of Technology and The University of Gothenburg, Gothenburg, Sweden; 4 Social Medicine, School of Public Health and Community Medicine, Institute of Medicine, The Sahlgrenska Academy at University of Gothenburg, Gothenburg, Sweden; 5 Department of Sociology and Work Science, University of Gothenburg, Gothenburg, Sweden

**Keywords:** Mixed-effects models, Time series analysis, Process evaluation, Sickness absence, Employee turnover, Workplace interventions, Organisation, Work environment, Public sector, Organisational-level intervention

## Abstract

**Background:**

Theoretical frameworks have recommended organisational-level interventions to decrease employee withdrawal behaviours such as sickness absence and employee turnover. However, evaluation of such interventions has produced inconclusive results. The aim of this study was to investigate if mixed-effects models in combination with time series analysis, process evaluation, and reference group comparisons could be used for evaluating the effects of an organisational-level intervention on employee withdrawal behaviour.

**Methods:**

Monthly data on employee withdrawal behaviours (sickness absence, employee turnover, employment rate, and unpaid leave) were collected for 58 consecutive months (before and after the intervention) for intervention and reference groups. In total, eight intervention groups with a total of 1600 employees participated in the intervention. Process evaluation data were collected by process facilitators from the intervention team. Overall intervention effects were assessed using mixed-effects models with an AR (1) covariance structure for the repeated measurements and time as fixed effect. Intervention effects for each intervention group were assessed using time series analysis. Finally, results were compared descriptively with data from process evaluation and reference groups to disentangle the organisational-level intervention effects from other simultaneous effects.

**Results:**

All measures of employee withdrawal behaviour indicated statistically significant time trends and seasonal variability. Applying these methods to an organisational-level intervention resulted in an overall decrease in employee withdrawal behaviour. Meanwhile, the intervention effects varied greatly between intervention groups, highlighting the need to perform analyses at multiple levels to obtain a full understanding. Results also indicated that possible delayed intervention effects must be considered and that data from process evaluation and reference group comparisons were vital for disentangling the intervention effects from other simultaneous effects.

**Conclusions:**

When analysing the effects of an intervention, time trends, seasonal variability, and other changes in the work environment must be considered. The use of mixed-effects models in combination with time series analysis, process evaluation, and reference groups is a promising way to improve the evaluation of organisational-level interventions that can easily be adopted by others.

## Introduction

Like other countries, Sweden is struggling to combat future labour shortages in the healthcare sector. About 120,000 new employees will be needed by 2026 to meet the needs of a growing population and an increasing proportion of retirees (SALAR 2018). An urgent demand for nurses, particularly specialist nurses, is anticipated to last, and the relatively balanced supply of physicians depends on the influx of 500 immigrant physicians yearly up to 2035 (Statistics Sweden 2017). Meanwhile, the Swedish healthcare sector is struggling with higher levels of sickness absence (SSIA 2018), with depression, anxiety, and adjustment disorders being the most frequent diagnoses (SSIA 2016). These common mental disorders are associated with poor psychosocial working conditions such as high job demands, low job control, and low social support (Aronsson et al. 2017; Vries et al. 2018; Stansfeld and Candy 2006). The combination of high demands and low decision authority has been found to be particularly common in the healthcare sector, and these factors have developed negatively in recent decades in Sweden (Cerdas et al. 2019).

One way for employers to attract new employees to healthcare professions is to ensure a sound work environment. To do so, it is equally important for the existing employees to thrive at work and for the employers to prevent employees from leaving the healthcare sector.

“(Non)attendance behaviours” (Daouk-Öyry et al. 2014) or “employee withdrawal behaviours” (Griffeth et al. 2000) such as absenteeism and leaving the job or profession altogether can all be seen as possible strategies by which employees cope with adverse working conditions (Josephson et al. 2008; Söderberg et al. 2014). Employee withdrawal behaviours in the healthcare system can seriously affect its overall performance in terms of, for example, care quality and financial outcomes (Gaudine and Gregory 2010; Homburg et al. 2009; Laschinger et al. 2009; Liu et al. 2012; McGillis Hall and Doran 2007). Although (non)attendance behaviours can be seen as resulting from interactions among factors at multiple levels (Daouk-Öyry et al. 2014), improving healthcare employees’ working conditions to reduce not only sickness absence but also other employee withdrawal behaviours should be made a priority in creating sustainable healthcare organisations (Josephson et al. 2008). While sickness absence and employee turnover are multifaceted phenomena that have been measured in various ways (Griffeth et al. 2000; Barak et al. 2001; Hensing 2004; Steel and Lounsbury 2009), other forms of employee withdrawal behaviour, such as unpaid leave or reduced working hours, have seldom been studied.

Organisational-level interventions have been recommended as an important but underutilised way to improve working conditions and address “the causes of the causes” of employee stress and ill-health, and their undesired organisational outcomes (Cox et al. 2007; Kompier 2001; Nielsen and Randall 2013; Nielsen et al. 2010a; Giga et al. 2003). However, the higher one climbs in the chain of events, the more analytical levels must be considered, since employees are nested in groups, which are nested in workplaces, which are grouped in organisations, and so on. In addition, organisations themselves are always embedded in even wider contexts (Johns 2006). From a realist viewpoint, the success of an intervention relies on the encounter between participating individuals and the resources provided by the intervention (Pawson et al. 1997). Whether or not these resources are used as planned by the participants depends on context-specific conditions. To be successful, an intervention must therefore fit its context. In contrast to an experimental design, which seeks to eliminate contaminating contextual factors to isolate specific mechanisms, a realist design instead sets out to examine what works for whom, under what circumstances.

It is widely recognised that concurrent changes in an organisation or its surroundings may affect an intervention and potentially interact with its effects (Grant and Wall 2009; Mills et al. 2006). Examples of such changes are concurrent organisational changes (Nielsen et al. 2006), other conflicting initiatives (Guastello 1993; Nielsen et al. 2010b), and macroeconomic changes (Nielsen and Abildgaard 2013). One methodological challenge when evaluating organisational-level interventions is to separate the effect of the intervention from the effects of other changes in the organisation and its surroundings.

In the past, evaluations of organisational-level interventions have shown that the effects of such interventions are inconclusive (Montano et al. 2014; Semmer 2006; Ruotsalainen et al. 2015; Gray et al. 2019). To understand these inconsistencies, it has been suggested that qualitative process data about how and why an intervention does or does not work are valuable (Nielsen and Randall 2013; Egan et al. 2007; Kristensen 2005; Nielsen et al. 2010c). Hence, another methodological challenge is how to combine qualitative and quantitative data in mixed-methods designs to evaluate intervention effects (Nielsen and Abildgaard 2013; Greasley and Edwards 2015; Härenstam et al. 2019).

In addition to the methodological challenges described above, the need for statistical analyses enabling effect-size analysis and more detailed between-groups and within-group variation to be explored over time, such as multilevel analysis and latent growth curve-modelling, has also been stressed for better evaluating what works for whom, and for how long (Burgess et al. 2020).

In 2017, an organisational-level intervention was launched in a large Swedish healthcare organisation to decrease sickness absence among employees. The intervention was designed to address organisational-level causes of the problems, rather than employee behaviours. This large-scale intervention was used to demonstrate how an organisational-level intervention evaluation can be designed to evaluate the overall effect on employee withdrawal behaviour of an organisational-level intervention that goes beyond experimental designs to better take context into account (Ruotsalainen et al. 2015; Guyatt et al. 1995; Richardson and Rothstein 2008).

## Aim

The study aims to investigate if mixed-effects models in combination with time series analysis, process evaluation, and reference group comparisons could be used for evaluating the effects of an organisational-level intervention on employee withdrawal behaviour. Using this methodological approach in evaluating a large-scale intervention, both methodological and practical implications of the implementation, evaluation, and result interpretation can be demonstrated and discussed.

## Methods

### Setting and study population

**Fig. 1:**
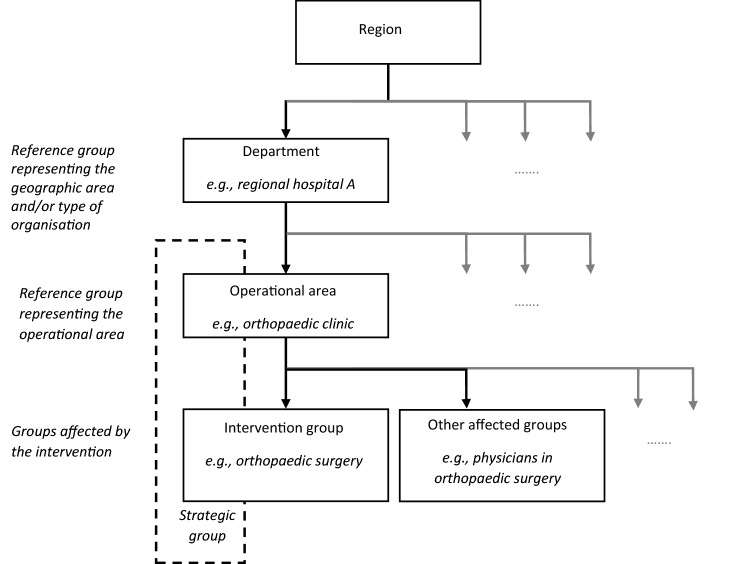
Schematic overview of the organisational levels in the region, including intervention groups, other affected groups, operational areas, and departments using intervention group 1 as an example; the organisational levels included in the strategic group have been marked in the figure

In Sweden, hospital and primary healthcare are managed by 21 regions. The studied intervention was carried out in a region with approximately 55,000 public-sector employees, of whom about 85% worked in the healthcare sector. The intervention was initiated and funded in 2017 as part of a political initiative to decrease the region’s sickness absence. Initially, eight operational areas (e.g., paediatrics and hospital service and maintenance) in five departments (i.e., four hospitals and one service department; see Fig. 1) were identified by the research team as having high sickness absence (> 10%, chosen pragmatically, the regions’ average total sickness absence varied between 5.5% and 6.8% from 2013 to 2019) in combination with high employee turnover (data not shown). These operational areas were approached and invited to participate in the intervention. Since it was impossible to include entire operational areas in the intervention, subgroups (i.e., the eight intervention groups, see Fig. 1) were selected in consultation with local managers and their HR partners, using their knowledge of the organisation (Table 1). Together, the eight selected groups comprised about 1600 employees.

**Table 1:** Background information about the eight intervention groups, their corresponding reference groups and information about the implemented measures and fidelity to the intention underlying the intervention

	Intervention group							
	1	2	3	4	5	6	7	8
Type of workplace	Orthopaedic surgery ward	Psychiatric hospital ward	Paediatric hospital clinic	Hospital service and maintenance unit	Paediatric hospital ward	Forensic psychiatric unit	Orthopaedic aid and sterilization technical unit	Radiology hospital unit
Mean number of employees (*n*, range)^a^	99 (90–105)	41 (29–49)	191 (182–204)	289 (261–311)	164 (88–199)	268 (242–281)	131 (108–146)	458 (443–471)
Mean percentage of men in the workplace (%)^a^	8	10	11	30	2	43	31	21
Start of the intervention (month, year)^b^	Feb, 2018	Oct, 2017	Sept, 2018	June, 2017	Oct, 2017	Jan, 2018	Aug, 2018	Oct, 2017
Fidelity (high/low)	High	High	High	Low	High	High	High	High
- Action plan was set up (yes/no)	Yes	Yes	Yes	No	Yes	Yes	Yes	Yes
- Action plans and actions target organizational conditions (yes/no)	Yes	Yes	Yes	No^c^	Yes	Yes	Yes	Yes
- Goal achievement (yes/no)	Yes	Yes	Yes	No	Yes	Yes	No	Yes
- Shared understanding and sustainable work processes (yes/no)	Yes	Yes	Yes	No	Yes	Yes	Yes	Yes
Performed measures under the intervention	- Workshop on work culture - Support action plan implementation - Coaching sessions for managers - Ergonomic assessments	- Workshop on improving work conditions - Support action plan implementation - Coaching sessions for managers and management board	- Occupational reflection groups - Coaching sessions for management board - Workshop on work environment for strategic group	- Coaching sessions for managers and management boards - Analysis of organisational conditions - Workshops and training	- Analysis of organisational conditions - Training for mentors - Work environment training for managers - Training in work practices for employees	- Analysis of organisational conditions - Coaching sessions for management board - Workshop on work culture	- Coaching sessions for managers - Work environment training for employees - Workshop on roles and responsibility	- Coaching sessions for managers and management board - Workshop on work environment for management board - Analysis of organisational conditions - Training for employees
Mean number of employees in the operational area (*n*, range)^a,d^	113 (106–121)	227 (199–254)	1440 (1400–1475)	-^e^	2404 (2261–2543)	3179 (3009–3280)	2020 (1977–2091)	1296 (1296–1363)
Mean number of employees in the department (*n*, range)^a,d^	460 (442–485)	1319 (1199–1405)	4532 (4444–4639)	2020 (1896–2290)	15,125 (14,582–15,589)	15,038 (14,431–15,562)	15,176 (14,590–15,680)	14,849 (14,229–15,347)
Identified unit affected but not included in the intervention (yes/no)	Yes	Yes	No	Yes	Yes	No	No	No

^a^Mean employees during the study (58 months, January 2015–October 2019)

^b^The month when the first measure affecting the employees was implemented

^c^Organisational measures not included in this intervention, though a larger separate reorganization was performed at the same time as the intervention

^d^ Employees belonging to the intervention group and other groups affected by the intervention not included

^e^Intervention group was the operational area

### Intervention design

First, external process facilitators were assigned to each intervention group to improve implementation and fidelity, or adherence, to the intention underlying the intervention (Härenstam et al. 2019; Augustsson et al. 2015). Second, a strategic group consisting of managers and their HR partners at two or more hierarchical levels was formed (see Fig. 1). The role of the strategic group was to identify group-specific causes of employee withdrawal behaviour, suggest measures to address these causes, and implement the suggested measures (Härenstam et al. 2019; Biron et al. 2010; Devos et al. 2007). The active involvement of the strategic group was also supposed to ensure a good fit between the interventional measures and the local context (McFillen et al. 2013; Nielsen et al. 2015).

**Fig. 2:**
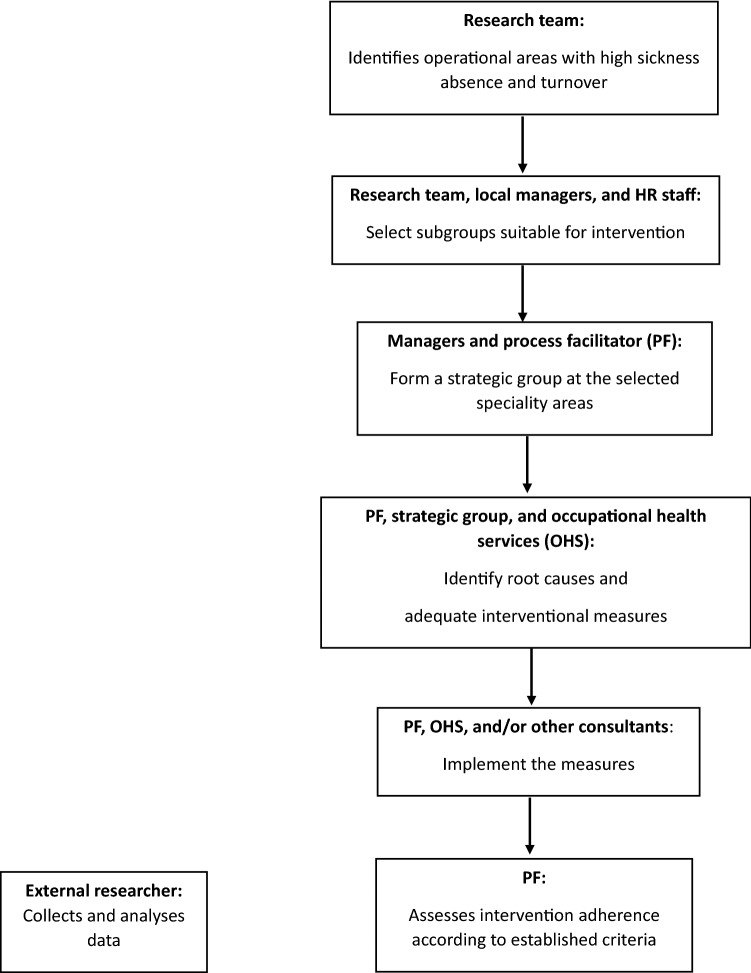
Overview of the intervention process

Interventional measures were intended to affect the employees’ work environment, preferably by targeting the “causes of the causes”, i.e., how work was organised and/or executed, rather than strengthening individual employees (Cox et al. 2007; Kompier 2001; Nielsen et al. 2010a; Johns 2006; Nielsen and Abildgaard 2013). The measures (Table 1) were implemented by the process facilitator, the region’s internal occupational health service, or external consultants. The intervention process and effects were evaluated as an externally funded project, separate from the intervention (see Fig. 2 for an overview of the intervention), since the task of evaluating the intervention was assigned after the intervention was launched.

In realist terms, the intervention program provided the workplaces with several resources. It financed the external process facilitators who assisted the strategic groups with expertise on occupational health and change management in the process of identifying and suggesting organisational-level measures, tailored to the specific issues and conditions at each workplace. The program also financed these interventional measures. The measures were designed to improve the work environment and trigger mechanisms that would lead to middle- or long-term outcomes such as improved work satisfaction and health among the employees. As an example, the strategic group of a hospital ward concluded that the operational managers needed professional help from an occupational psychologist to reorganise their respective units to strengthen inter-unit collaboration and workflow (the measure). The improved workflows resulted in a decreased workload (middle-term effect) and reduced stress (Semmer et al. 2007) among the employees (the mechanism). With time, they became less prone to leave their workplaces (long-term outcomes). However, whether the mechanism is trigged or not depends on how well the measure is tailored to and received at the specific workplace. In other words, the effect of each measure depends on context. Since all measures were different, the assumed chain of events was specific for all intervention groups.

### Data collection

The data collection and the statistical analyses of employee withdrawal behaviour were mainly performed by a project member (first author) not involved in the initiation, planning, or implementation of the intervention.

To separate the effect of the intervention from the overall development in each corresponding operational area and department during the time of the intervention (Grant and Wall 2009; Mills et al. 2006), data were also collected for these two organisational levels (minus the groups affected by the intervention; Fig. 1 and Table 1). Four of the eight intervention groups had another unit or group of employees that was affected by, but not part of, the intervention measures. Examples of such groups were physicians serving in a medical ward who participated in the intervention and medical or administrative units sharing the same patient groups or tasks. Data for these groups were also retrieved.

### Measures of effect on employee withdrawal behaviour

Monthly data on sickness absence, employment rate, unpaid leave, and employee turnover between January 2015 and October 2019 were retrieved from the region’s employee administration system. *Sickness absence* was calculated as the percentage absence at a group level based on the number of hours of absence due to sickness divided by the total number of hours the group was expected to work each month (minus vacation time, parental leave, and leave to care for sick children). The data were also stratified by short-term (1–14 days) and long-term sickness absence (> 60 days); sickness absence lasting 1–14 days was also expressed in the number of absence days per employee. *Employee turnover* was expressed in the percentage turnover (i.e., number of individuals leaving the workplace divided by the total number of employees in each group and month) and was stratified by those leaving for another position within the region and those leaving for employers outside the region. *Employment rate* was calculated as the average percentage of working hours relative to full time for each group and month. Finally, *unpaid leave* was assessed as the number of absence days without payment (expressed in days of absence per employee).

### Process evaluation

To include a process evaluation aspect in the effect evaluation (Nielsen and Randall 2013; Augustsson et al. 2015), fidelity to the intention underlying the intervention was assessed using the criteria of Härenstam et al. (Härenstam et al. 2019). Their four criteria were adapted to the study context and formulated as whether: (1) the strategic group formulated action plans and began to implement them; (2) the action plans contained measures intended to affect the employees’ work environment, preferably by targeting how work was organised and/or executed rather than by strengthening the individual employees; (3) the measures in the action plan were implemented and the expected results were at least partially achieved during the implementation phase; and (4) the intervention led to a shared understanding and a sustainable work process in the intervention group.

These criteria were assessed for each intervention group by the external process facilitators, using their knowledge of the entire process (i.e., initiation, screening, action planning, and implementation) in structured group interviews led by a project member (i.e., the first author). The results of the assessments were then used to determine low versus high fidelity (Table 1). The assessments were performed before the process facilitators were informed of the results of the study. After the assessments, the process facilitators’ qualitative reflections on the results for each intervention group were also collected and used to identify other conflicting initiatives and the overall interpretation of the results (see below).

### Statistics

The measures of employee withdrawal behaviour were tested for normality using the Shapiro–Wilks test and visual inspection of the generated histograms. An assumption of normality was assessed to be plausible, and the parametric methods were used on untransformed data in the subsequent analyses.

The seasonal variation among the measures was visualised by calculating means of group-specific means (of the 4–5 years of available data) for each month. The variability in the employee withdrawal behaviour measures was estimated using simple mixed-effects models with a random intercept and with or without a random slope, with time (to control for time trends), year (continuous), and month (categorical 1–12) as fixed effects. Using the variance components of the random-intercept model, the intra-class correlation (ICC = σ^2^^_bY_ / σ^2^^_Y_) was estimated to investigate how much of the variation could be explained by the variation between groups. Statistical significance was determined at *p* < 0.05, and two-sided confidence intervals were calculated.

The intervention effects were evaluated in three steps. In step 1, overall effects were estimated for the intervention groups, and any concurrent effects for the reference groups were also determined using a random-intercept or random-coefficient model (PROC MIXED in SAS version 9.4; SAS Institute, Cary, NC, USA) with group and time (nested within group) as random effects. In addition, a first-order autoregressive correlation structure (AR[1]) was used to account for correlations between repeated measurements of the same group. Fixed effects for year (continuous) and month (categorical 1–12) were added to the model to control for time trends and seasonality, and a dummy variable for the intervention (0 up to the beginning of the intervention and then 1; Table 1) was added to analyse the effect of the intervention. Interaction terms between the intervention variable and intervention group, fidelity (high/low), and time (continuous), respectively, were also added to investigate the differences in the intervention effects between groups and changes over time. To investigate delayed intervention effects, an intervention effect with a time lag of 1, 3, or 6 months after the start of the intervention was added to the models. Hypothesis testing for fixed effects was performed using Wald tests, and tests of random effects were performed using likelihood ratio tests.

In step 2, the potential effects of the intervention on the intervention groups and their respective reference groups were estimated using Box–Jenkins autoregressive integrated moving average (ARIMA) time series methodology (Box and Jenkins 1976; Tabaschnick and Fidell 2013) to discover whether the intervention effect size and/or direction differed between groups within the intervention. An ARIMA model including seasonal components was derived for each measure and group using the Time Series Modeler in SPSS Statistics version 25 (IBM, Armonk, New York, USA). The intervention variable was then added to these models to analyse the effect of the intervention. An ARIMA model containing a first-order autoregressive element, a seasonal effect, and/or a first-order difference representing the lingering effect was found to best represent the time series data for most measures of employee withdrawal behaviour except for employee turnover, which often lacked a time trend in the data.

In the third and final step, the estimated intervention effects (both overall and for individual intervention groups) were compared with the results of the separate analyses of the reference groups (Fig. 1) to identify any plausible explanations other than the intervention for the estimated intervention effects. This was done by comparing the effect sizes and directions of the estimated intervention effects in the intervention and reference groups and by comparing these with the results of the process evaluation.

## Results

### Variability in employee withdrawal behaviour, and associations with time

**Fig. 3:**
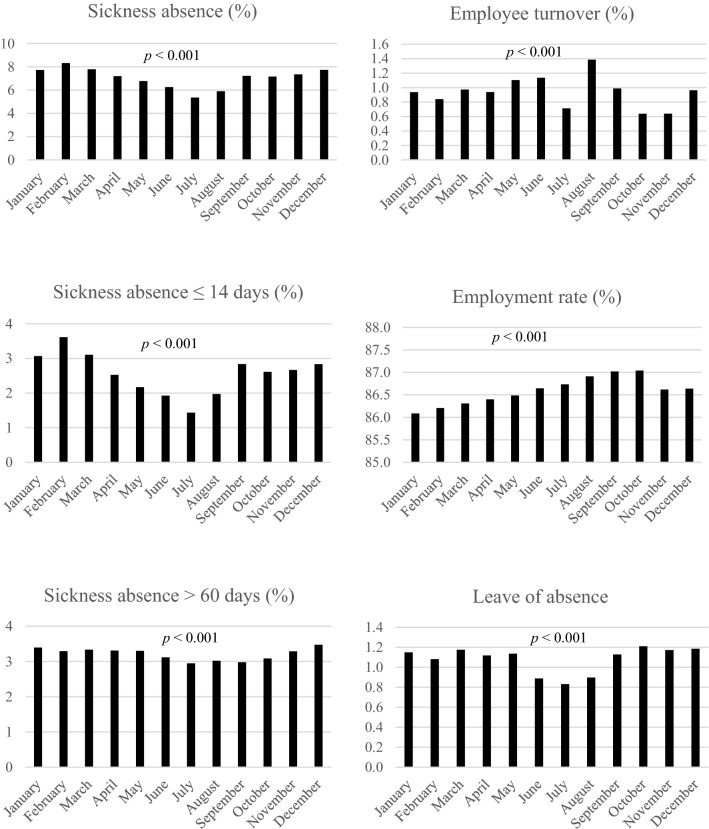
Seasonal variability, mean of individual group means per month, in sickness absence, employee turnover, and other types of absences for the eight departments (intervention groups excluded)

The seasonal variability in employee withdrawal behaviour was illustrated by computing monthly means of the individual group means for the departments (intervention groups excluded) during the study (Fig. 3). For the departments and operational areas, all measures of employee withdrawal behaviour were found to have significant (*p* < 0.001) seasonal variability when assessed in a mixed-effects model with time as fixed effect (Fig. 3). In the intervention groups, seasonal variability was seen in sickness absence (*p* < 0.001 for total, ≤ 14 days in %, and ≤ 14 days in days/employee and *p* = 0.04 for > 60 days), employee turnover to external employer (*p* = 0.02), and unpaid leave (*p* < 0.001).

Time trends in sickness absence, employment rate, unpaid leave, and employee turnover were investigated using a mixed-effects model with time as fixed effect. Since 2015, a decrease of about 0.13% annually in the total sickness absence (*β* =  − 0.13, 95% CI − 0.42 to − 0.018, *p* = 0.03) and an increased employee turnover of about 0.02% annually (*β* = 0.022, 95% CI 0.0055–0.039, *p* = 0.02 for total turnover and *β* = 0.024, 95% CI 0.0097–0.039, *p* = 0.006 for turnover to external employer) were seen in the departments (intervention groups excluded). Increased employment rates were also seen, both in the departments (*β* = 1.1, 95% CI 0.83–1.4, *p* < 0.001) and within the respective operational areas (*β* = 1.4, 95% CI 0.73–2.0, *p* = 0.002). However, the intervention groups displayed no overall time trend in sickness absence or employment turnover during the study period.

Between 30 and 50% of the total variability in sickness absence and 10–20% of the employment turnover were explained by the between-group variability among the groups with high sickness absence. The proportion of between-group variability increased somewhat when the models were corrected for time trends in data (Table 2). For employment rate, almost all variability was attributed to the between-group variability. When correcting for the time trend in data, the within-group variability decreased by an average of about 16% (1.2–39%), with the largest decrease being found for sickness absence ≤ 14 days, i.e.,  – 39% expressed in percent and  – 33% expressed in days/employee (Table 2).

**Table 2:** Within- and between-group variability of employee withdrawal behaviour

	Samples^a^	Subjects^a^	No fixed effects^b^				Fixed effects: year and month^b^			
			σ^2^^_Y_	σ^2^^_bY_	σ^2^^_wY_	ICC^c^	σ^2^^_Y_	σ^2^^_bY_	σ^2^^_wY_	ICC^c^
Sickness absence (%)	289	8	10.41	3.47	6.94	0.33	9.30	3.65	5.65	0.39
Sickness absence ≤ 14 days (%)	289	8	1.78	0.72	1.06	0.40	1.38	0.74	0.65	0.53
Sickness absence ≤ 14 days (days/employee)	289	8	0.11	0.05	0.06	0.49	0.09	0.05	0.04	0.59
Sickness absence > 60 days (%)	289	8	5.36	1.96	3.40	0.37	5.17	1.92	3.25	0.37
Employee turnover to external employer (%)	289	8	1.01	0.12	0.90	0.11	0.98	0.12	0.86	0.13
Employee turnover within region (%)	289	8	0.36	0.04	0.32	0.11	0.37	0.04	0.33	0.11
Employment rate (%)	289	8	97.42	93.98	3.44	0.96	96.62	93.98	2.65	0.97
Unpaid leave (days/employee)	289	8	0.22	0.10	0.11	0.48	0.20	0.10	0.10	0.52

### Estimation of overall intervention effects on employee withdrawal behaviour

An overall positive effect, in terms of decreased sickness absence, was found for total sickness absence (a decrease of 2 percentage points, *β* =  − 1.9, 95% CI − 2.8 to − 0.89, *p* < 0.001) and sickness absence ≤ 14 days (a decrease of 0.08 days/employee, *β* =  − 0.08, 95% CI − 0.14 to − 0.02, *p* = 0.01; Table 3). For employee turnover, employment rate, or unpaid leave, no overall effects were seen (Table 3). Furthermore, the long-term sickness absence (> 60 days) in the studied departments increased by 0.14 percentage points during the time of the intervention (*β* = 0.14, 95% CI 0.01–0.26, *p* = 0.03), which was not reflected in the intervention groups. No other simultaneous effects were detected in the respective operational areas or departments. Also, no effects were seen in the groups affected by, but not part of, the intervention (Table 3).

**Table 3:** Overall intervention effects on employee withdrawal behaviour

		Intervention group	Other affected group	Operational area^a^	Department^a^
Sickness absence (%)	*β* (95% CI)	– 1.9 (– 2.8 to – 0.89)	0.68 ( – 1.2 to 2.5)	0.03 ( – 0.38 to 0.44)	0.08 ( – 0.14 to 0.30)
	*p* value	< 0.001	0.5	0.9	0.5
Sickness absence ≤ 14 days (%)	*β* (95% CI)	– 0.11 – 0.39 to – 0.16)	0.09 ( – 1.5 to 1.6)	0.07 ( – 0.05 to 0.20)	0.03 ( – 0.06 to 0.12)
	*p* value	0.4	0.9	0.3	0.5
Sickness absence ≤ 14 days (days/employee)	*β* (95% CI)	– 0.08 (–0.14 to – 0.02)	0.05 ( – 0.06 to 0.16)	0.01( – 0.02 to 0.04)	– 0.008 ( – 0.03 to 0.02)
	*p* value	0.01	0.3	0.5	0.5
Sickness absence > 60 days (%)	*β* (95% CI)	0.03 (–0.72 to 0.78)	– 2.2 (–5.9 to –1.6)	0.01 (–0.16 to 0.40)	0.14 (0.01 to 0.26)
	*p* value	0.9	0.3	0.4	0.03
Employee turnover (%)	*β* (95% CI)	– 0.28 (–0.65 to 0.09)	0.10 ( – 0.66 to 0.86)	0.03 ( – 0.16 to 0.22)	– 0.05 ( – 0.12 to 0.03)
	*p* value	0.1	0.8	0.8	0.2
Employee turnover to external employer (%)	*β* (95% CI)	– 0.18 ( – 0.48 to 0.13)	0.05 ( – 0.48 to 0.57)	0.04 ( – 0.10 to 0.19)	– 0.02 ( – 0.08 to 0.03)
	*p* value	0.3	0.9	0.5	0.4
Employee turnover within region (%)	*β* (95% CI)	– 0.05 (–0.23 to 0.14)	0.15 ( – 0.38 to 0.70)	– 0.02 (–0.13 to 0.09)	– 0.006 (–0.05 to 0.04)
	*p* value	0.6	0.6	0.7	0.8
Employment rate (%)	*β* (95% CI)	0.42 (–0.10 to 0.93)	0.11 ( – 1.4 to 1.6)	– 0.01 ( – 0.35 to 0.33)	0.002 ( – 0.44 to 0.15)
	*p* value	0.1	0.9	0.9	1.0
Unpaid leave (days/employee)	*β* (95% CI)	0.02 ( – 0.11 to 0.15)	– 0.10 ( – 0.49 to 0.29)	– 0.01 ( – 0.09 to 0.07)	0.03 ( – 0.02 to 0.08)
	*p* value	0.8	0.6	0.8	0.3

^a^Intervention group and other groups affected by the intervention excluded

### Estimation of delayed intervention effects

When adding an intervention effect at a time lag of 1, 3, or 6 months to the model, the decreased total sickness absence persisted for the 1- and 6-month time lags, but the decrease in short-term sickness absence, expressed in days per employee, only persisted for the 1-month time lag and no statistically significant intervention effect was seen for the 3- or 6-month time lag (data not shown). The addition of time lags also revealed a statistically significant decreased employee turnover of 0.4 percentage points for both total turnover (*β* =  − 0.43, 95% CI − 0.79 to − 0.073, *p* = 0.02 for the 1-month time lag, *β* =  − 0.37, 95% CI − 0.71 to − 0.025, *p* = 0.04 for the 3-month time lag, and *β* =  − 0.43, 95% CI − 0.75 to − 0.011, *p* = 0.009 for the 6-month time lag) and turnover to external employer (data not shown).

### Estimation of factors affecting the intervention effect

Factors affecting the intervention effect were investigated by adding interaction effects between the intervention variable (yes/no) and intervention group, fidelity (high/low), and time (continuous) after the intervention, respectively. There was a statistically significant interaction effect between the intervention variable and intervention group for sickness absence, internal employee turnover, employment rate, and unpaid leave (*p* < 0.001 for all, except *p* = 0.07 for total sickness absence), revealing that the intervention effect varied between intervention groups (see below). Seven of the eight intervention groups were regarded as having high fidelity to the intention underlying the intervention (all except intervention group 4; Table 1). For short-term sickness absence, a statistically significant interaction effect (*p* < 0.001) was found between the intervention variable and fidelity (intervention effect, stratified by fidelity, in %: *β* = 0.06, 95% CI − 0.63 to 0.75, *p* = 0.9 for low fidelity and *β* =  − 0.16, 95% CI − 0.46 to 0.15, *p* = 0.3 for high fidelity and in days/employee: *β* =  − 0.02, 95% CI − 0.18 to 0.14, *p* = 0.8 for low fidelity and *β* =  − 0.08, 95% CI − 0.02 to − 0.15, *p* = 0.009 for high fidelity) but not for the other measures of employee withdrawal behaviour.

When adding an interaction effect between the intervention variable and time to the model, a statistically significant increase in the intervention effect with time was seen for total sickness absence, with a 1% increase in the intervention effect per year after the intervention (*β* =  − 1.0, 95% CI − 1.9 to − 0.12, *p* = 0.03), and for total employee turnover and turnover to external employer, with a 0.4% increase in the intervention effect per year (*β* =  − 0.39, 95% CI − 0.73 to − 0.063, *p* = 0.02 for total employee turnover and *β* =  − 0.36, 95% CI − 0.61 to − 0.11, *p* = 0.005 for turnover to external employer). The inclusion of an interaction effect between the intervention variable and time somewhat increased the estimate of the intervention effect (*β* =  − 2.5, 95% CI − 6.5 to 1.5, *p* = 0.22 for total sickness absence, *β* =  − 1.5, 95% CI − 3.0 to 0.043, *p* = 0.06 for total employee turnover, and *β* =  − 1.4, 95% CI − 2.6 to − 0.29, *p* = 0.01 for turnover to external employer).

### Estimation of group-specific intervention effects on employee withdrawal behaviour

According to the process evaluation, all intervention groups gained some positive effects related to the identified cause of their problems, such as increased awareness, working tools for the future, and increased internal collaboration. A positive effect on sickness absence and/or employee turnover could be seen for three of the eight intervention groups (groups 2, 4, and 8; Table 4). In addition, for two groups (groups 1 and 7), both the operational area and the department had increased total sickness absence, in per cent (group 1, *β* = 1.6, *p* = 0.07 and *β* = 1.6, *p* = 0.03, respectively), and increased long-term sickness absence, in per cent (group 7, *β* = 0.22, *p* = 0.07 and *β* = 0.14, *p* = 0.03, respectively), without any increase in the intervention group; at least for group 1, this was assessed by the process facilitators to be an intervention effect.

**Table 4:** Effects of the intervention on the employment withdrawal behaviour of individual intervention groups

		Intervention group							
		1	2	3	4	5	6	7	8
Sickness absence (%)	*β*	– 2.2	– 11.2	0.80	– 2.2	– 0.84	– 0.83	– 0.57	– 0.96
	*p* value	0.2	< 0.001	0.4	0.004	0.2	0.2	0.5	0.01
Sickness absence ≤ 14 days (%)	*β*	– 0.040	– 0.40	– 0.24	– 0.51	0.33	– 0.14	– 0.15	0.009
	*p* value	1.0	0.5	0.2	0.1	0.4	0.6	0.6	1.0
Sickness absence ≤ 14 days (days/employee)	*β*	0.15	– 0.11	– 0.059	– 0.010	0.036	0.008	0.004	0.037
	*p* value	0.02	0.3	0.3	0.9	0.6	0.9	1.0	0.3
Sickness absence > 60 days (%)	*β*	0.037	2.6	0.29	– 1.6	– 0.42	– 0.29	0.27	– 0.19
	*p* value	0.9	0.2	0.6	< 0.001	0.6	0.4	0.7	0.5
Employee turnover (%)	*β*	0.17	– 1.1	0.068	0.29	0.30	0.17	0.53	0.22
	*p* value	0.6	0.07	0.8	0.4	0.2	0.7	0.06	0.1
Employee turnover to external employer (%)	*β*	0.014	– 0.99	0.20	0.28	0.28	– 0.077	0.51	0.21
	*p* value	1.0	0.04	0.3	0.4	0.1	0.7	0.07	0.1
Employee turnover within region (%)	*β*	0.15	– 0.078	– 0.13	0.003	0.023	0.13	0.017	0.020
	*p* value	0.4	0.8	0.2	0.9	0.8	0.09	0.8	0.5
Employment rate (%)	*β*	– 0.12	4.9	0.19	0.24	– 0.96	– 0.73	– 0.98	– 0.096
	*p* value	0.8	< 0.001	0.8	0.4	0.4	0.02	0.8	0.6
Unpaid leave (days/employee)	*β*	– 0.12	– 0.12	0.063	– 0.20	– 1.70E–05	0.25	– 0.12	– 0.062
	*p* value	0.5	0.6	0.5	0.2	1.0	0.06	0.6	0.5

However, simultaneous statistically significant effects (both positive and negative) on sickness absence and/or employee turnover were seen in all operational areas and departments during the time of the intervention (data not shown). When adding the information from the process evaluation and analyses of the operational areas and departments, two of the three intervention groups with a statistically significant positive effect (groups 4 and 8) had plausible alternative explanations for the positive results other than the intervention. Intervention group 4 performed a large reorganisation in parallel with the intervention, which probably improved the work environment. For intervention group 8, a decrease in sickness absence was seen in both the intervention group and the two reference groups, which was assessed as probably caused by changes throughout the operational area and/or department rather than by the intervention.

When adding a lagged intervention effect at 1, 3, or 6 months, the overall result persisted, though there was a tendency for a decreased positive effect on sickness absence (data not shown).

## Discussion

This study answers the call from scholars to develop improved mixed methods for the evaluation of organisational-level interventions. This novel methodological approach, combining mixed-effects models with time series analysis, process evaluation, and reference group comparison, responds to the methodological challenges of better taking the context into account and separating the intervention effects in organisation-level interventions from other workplace changes (Nielsen and Randall 2013; Gray et al. 2019; Greasley and Edwards 2015; Härenstam et al. 2019). This approach enables the investigation of both overall and context-specific intervention effects, while enabling some understanding of the mechanisms involved.

To better evaluate the total effect of organisational-level interventions on employee withdrawal behaviour, register data on a broad range of employee withdrawal behaviours were analysed (Daouk-Öyry et al. 2014; Griffeth et al. 2000). The results indicated that all these measures had statistically significant seasonal variations. In addition, time trends were also identified in the data for the respective operational areas and departments, revealing generally decreasing sickness absence and increasing employee turnover during the study period. These findings point to the importance of taking account of variation and trends in the effect measures (Lidwall and Marklund 2011). To prevent seasonal variations and time trends from affecting the evaluation of the intervention effects, the non-stationary in data must be accounted for in the models by adding year and month as fixed effects. In our case, the total variability decreased by an average of 16% (1.2–39%) when year and month were added to the model. Due to the pronounced seasonal variability, short-term sickness absence was affected more than the other measures; this must be considered when designing a study to evaluate the effects of organisational-level interventions over time.

Another challenge when analysing time series data is autocorrelation in repeated measurements over time (Zeger et al. 2006), with our analyses showing that a first-order autoregressive correlation structure could successfully be used to account for correlations between repeated measurements of employee withdrawal behaviours in the same group.

Applying this suggested methodological approach when evaluating the effects of an organisational-level intervention resulted in an estimated overall decrease in total sickness absence of about 2 percentage points. When sickness absence was stratified by duration, an effect on the short-term sickness absence was seen when expressed in sickness absence days per employee but not when expressed in percentage absence. Initially, no significant intervention effects were seen for employee turnover. However, a delayed decreased overall effect on total employee turnover and turnover to external employer was estimated when adding an intervention effect with a time lag of 1, 3, or 6 months to the model, highlighting the need to account for the time starting from deciding to change jobs and extending to leaving the workplace and becoming registered in the employer’s employee administrative system. The positive overall effects on sickness absence and employee turnover are well in line with the results of previous organisational-intervention studies (Bond and Bunce 2001; Framke et al. 2016; Lavoie-Tremblay et al. 2005; Munz et al. 2001).

To rule out other competing explanations for the estimated positive overall effects on employee withdrawal behaviour, simultaneous changes within the respective operational areas and departments were investigated. These analyses revealed no competing explanations for the positive overall intervention effects. However, an increase in long-term sickness absence was noted at the department level during the intervention, an increase not seen in the intervention groups. This finding underscores the importance of conducting comparative analyses (Grant and Wall 2009; Mills et al. 2006), since even a “non-effect” can prove positive in comparison.

Despite the broad range of employee withdrawal behaviours investigated here, intervention effects were mainly seen for total sickness absence and total employee turnover. One possible explanation for the absence of significant effects on the other measures of employee withdrawal behaviour might be the limited number of events gauged by these measures among the 1600 employees affected by the intervention. Despite this, we still believe that including a wider range of measures of employee withdrawal behaviour, especially for larger study groups, might provide important knowledge when evaluating organisational-level workplace interventions due to the known complexity and challenges of using sickness absence and employee turnover as measures (Griffeth et al. 2000; Barak et al. 2001; Hensing 2004; Steel and Lounsbury 2009).

The need for qualitative process data about how and why interventions do or do not work has previously been stressed (Nielsen and Randall 2013; Egan et al. 2007; Kristensen 2005; Nielsen et al. 2010c). Combining process evaluation data with the evaluation of quantitative effect measures at multiple levels offers an opportunity to improve our knowledge of how and why interventions do or do not work. The need to analyse group-specific effects was also seen in the analyses of overall intervention effects, since significantly varying effects on sickness absence and employee turnover were found between the eight intervention groups. Our time series analyses of the individual intervention groups made it possible to reveal that the overall intervention effect concealed a more heterogeneous pattern of group-specific intervention effects, stressing the importance of the context where the intervention is initiated, planned, and implemented (Nielsen and Randall 2013; Egan et al. 2007; Kristensen 2005; Nielsen et al. 2010c).

Using this suggested methodological approach could also rule out potentially competing explanations for the estimated intervention effects at a group level. Initially, a positive intervention effect on total sickness absence and/or employer turnover was seen in five of the eight intervention groups. However, when analysing simultaneous effects among the respective operational areas and departments, statistically significant effects on sickness absence and/or employer turnover were seen in all reference groups. By comparing the estimated effects with the respective results for the respective reference groups and process evaluations, alternative explanations could be suggested for three of the five groups, once again highlighting the importance of conducting comparative analyses (Grant and Wall 2009; Mills et al. 2006) and process evaluations to obtain the full understanding (Nielsen and Randall 2013; Egan et al. 2007; Kristensen 2005; Nielsen et al. 2010c).

The large variation in intervention effects between intervention groups could be explained by differences in fidelity to the intention underlying the intervention. However, only one of eight groups was classified as having low fidelity and an effect of fidelity on the intervention effect was only seen for short-term sickness absence. Several other contextual aspects, such as differences in implementation and/or within the organisation, could plausibly affect the intervention group, making it a challenge to detect effects among smaller groups in a complex organisational context.

Using all data produced in the evaluation, conclusions could be drawn as to whether or not this organisational-level intervention was successful. Despite the large differences between the intervention groups, we believe that this organisational-level intervention was successful, as it had a positive overall effect on the long-term outcomes in the assumed causal chain depicted in the Introduction. It is less likely to find effects on long-term outcomes, than on outcomes that are more short- or middle-term to the interventional mechanism. Thus, even very small decreases of a few percentage points on long-term outcomes such as sickness absence and employee turnover can be considered to be unexpected and of practical relevance, since they are so hard to get at. The positive overall result of the intervention might be because the involvement of external process facilitators led to high fidelity to the intention underlying the intervention or because the involvement of the strategic group meant that the measures properly fit the context (McFillen et al. 2013; Nielsen et al. 2015).

A strength of this study was its access to employee withdrawal behaviour data from the employers’ employee administrative system, in contrast to the self-reported data commonly used in effect evaluations. The data also captured short-term sickness absence, which in Sweden is covered by the employer and thus cannot be retrieved from the official governmental registers often used in such studies. Another strength of this study was its use of trained process facilitators without previous connections to the intervention group, which reinforced fidelity to the intervention in the intervention groups. These facilitators also provided information, used in the process evaluation, about the planning and implementation context of the intervention in each intervention group. To limit the risk of bias, the information for the process evaluation was collected using structured group interviews with the process facilitators before they received information about the evaluation results.

A limitation of this study was that the effect measures were limited to different measures of employee withdrawal behaviour, which are long-term outcomes in the assumed causal chain depicted in the Introduction. To fully reveal the mechanisms involved, it would have been appropriate also to operationalise and measure both the mechanisms and middle-term outcomes that appear earlier in the chain of events, such as changes in working conditions, employee motivation, and job satisfaction (Kompier 2001; Corbière et al. 2009; Lange et al. 2003). It is plausible that the intervention would have a larger impact of such middle-term outcomes. To contribute more fully to the tradition of realist evaluation, the study could have gone further in opening the “black box”, and revealing the mechanisms by which the intervention operated (Salter and Kothari 2014). However, the task of evaluating the intervention was assigned after the intervention was launched, limiting the possibility of collecting the data needed for such an approach. Also, as in all complex organisational-level interventions, it is a challenge to balance methodological ideals against practical considerations. Relying on data generated in registers and by external process facilitators, the data collection of this study could be conducted with a minimal interference with the daily operations of the involved workplaces. This could be considered an advantage from the practitioners’ point of view.

Another limitation of this study was that we were limited to using reference groups at a higher organisational level than the intervention groups, since it was impossible to find matched control groups or to retrieve information about reference groups at the same organisational level as the intervention groups due to technical limitations in the regions’ administrative employee system. If reference groups at the same organisational level had been available, comparisons between intervention and reference groups made within the same models could have been used, instead of comparing the results of two or more separate models.

It is also worth mentioning that this intervention was implemented in workplaces identified as having high sickness absence in combination with high employee turnover. Improving such severe situations requires time, and the somewhat limited follow-up time of 13–28 months after intervention start might have been insufficient to capture the full intervention effect. In addition, the evaluated effect might be overestimated if compared to a reference group with low sickness absence due to regression to the mean (Barnett et al. 2005). Although the intervention groups were selected from operational areas with high sickness absence in combination with high employee turnover, the same operational areas also served as reference groups, thus reducing the risk of spurious effects due to regression to the mean.

## Conclusions

This study presents a promising novel methodological approach for improving the evaluation of organisational-level interventions addressing employee withdrawal behaviour, interventions that can easily be adopted by others.

Our results indicate that when analysing organisational-level intervention effects, time trends, seasonal variability, and other changes in the work environment should be considered. This can be done using mixed-effects models in combination with time series analysis, process evaluation, and reference group comparisons. The possibility of analysing both overall intervention effects and intervention effects on individual intervention groups helped open the “black box” of evaluating organisational-level interventions, providing important information about the mechanisms and context of the intervention.

Applying this mixed-methods approach to the evaluation of an organisational-level intervention in the Swedish public sector revealed an overall decrease in employee withdrawal behaviour. However, the results also revealed large variation in the intervention effect between individual intervention groups, variation that could be used to understand how and why the intervention did or did not work.

## Contributions to the literature

- Research has shown methodological challenges when evaluating the effects of organisational-level interventions to decrease employee withdrawal behaviour, such as sickness absence and employee turnover.
- We found that time trends, seasonal variability and other changes in the workplace affect the measures of employee withdrawal behaviour and must be considered when evaluating organisational-level interventions.
- This study presents a novel methodological approach, combining mixed-effects models with time series analysis, process evaluation, and reference group comparison, which responds to the methodological challenges of better taking the context into account and separating the intervention effects in organisation-level interventions from other workplace changes.
